# Gangrenous Tooth Pulps as Centers of Infection

**Published:** 1888-04

**Authors:** W. D. Miller

**Affiliations:** Berlin, Germany


### Gangrenous Tooth-Pulps as Centers of Infection.

PRELIMINARY COMMUNICATION BY W. D. MILLER, M.D., D.D.S.,

BERLIN, GERMANY.

[Read before the Mississippi Valley Dental Association, held at Cincinnati, O., March 7, 1888.]

The subject which I have in mind, not to discuss, but simply to propose to you for consideration or investigation, is one of very great interest to the dentist, as well as the general practitioner and bacteriologist. You are all familiar with the commonly accepted belief that pericementitis and alveolar abscess are in the great majority of cases caused by putrefying organic matter in the roots of teeth. It is equally well known that the same cause may give rise to much more serious results, among others ostitis, necrosis, pyæmia, septicæmia, etc., terminating not unfrequently fatally. It becomes therefore a matter of the greatest importance to obtain an insight into the character of the infection brought about by the agency of putrid gangrenous or suppurating tooth pulps, and of the micro-organisms associated with it.

It is a fact well known that pathogenic bacteria are frequently found in the human mouth (we might say, I think, *constantly*, particularly in unclean mouths) and a suppurating tooth pulp seems to act as a sort of sieve to separate them from the non-pathogenic, as I have not been able to find the great variety of bacteria, in tooth pulps, which I have found in the mouth. Moreover, the bacteria of such pulps, so far as my observation at present goes, appear to be, as a rule, more pathogenic than the bacteria of the healthy mouth.

The experiments which I have undertaken in connection with this subject, are little more than begun, and Dr. Wright is responsible for their being presented in their present unripe condition. I shall consequently attempt only to describe the methods of experimentation which I have adopted, and to give, at most, one or two of the results thus far obtained.

In order to secure portions of gangrenous pulps which might not be impurified by outside material, I proceeded as follows: The freshly extracted tooth was mechanically cleaned and brought for a moment into a solution of bichloride of mercury (5 to 1,000). It was then rinsed in a large quantity of sterilized water, in order to remove the sublimate, split with sterilized forceps and a portion of the putrid pulp removed, of course with a sterilized needle.

The material obtained in this way was used for inoculating mice subcutaneously, a pocket having been formed at the root of the tail for this purpose. Up to the present I have made 118 inoculations, followed, in a great majority of cases, in twenty-four hours by inflammation and swelling at the point of inoculation. At the end of the second or third day a small abscess will usually be found, which, if opened, evacuates a drop of pus densely impregnated with micro-organisms. The reaction is not so severe or extensive as it generally is when a human being is inoculated by forcing gangrenous pulp tissue through the apical foramen, for the reason that in the latter case the point of inoculation lies much deeper and is surrounded by bone tissue.

In the case of the twelfth pulp experimented with, the reaction was very interesting and characteristic. In twelve to fifteen hours a blackish or blueish-black spot had formed around the pocket; in twenty-four hours some swelling was noted and already a slight fluctuation, *i. e.*, a slight formation of pus. At the end of the second day a tumor about the size of a pea had developed, which, on being punctured, evacuated a considerable quantity of pus mixed with gas and emitting an exceedingly strong and offensive odor. A second mouse inoculated with a small quantity of this pus developed exactly the same symptoms; likewise a third inoculated from the second, and so on to the twelfth generation. At this time I was obliged to leave Berlin for a few days, during which the mouse last inoculated died, and with it this series of inoculations. The specific bacterium of this strictly gangrenous process, I was unable to cultivate for the reason that it, like many others of the oral bacteria, does not grow upon artificial media.

In two other cases the inoculation was followed by blood poisoning, the mice dying in from two to six days, with large num. bers of micro-organisms in the blood and organs. From these few experiments it may already be seen that each gangrenous tooth pulp may be in itself a centre of infection, or it may serve as a channel through which pathogenic bacteria from the oral cavity may invade the tissue surrounding the point of the root, or even obtain entrance into the circulation.

I propose, in the course of a few months, to report at length upon the experiments now in progress.

DISCUSSION.

*Dr. Atkinson* spoke of the septic condition of roots and the necessity of cleansing to and often beyond the apex. He stated how uric acid accumulated, and the necessity of its speedy elimination from the system.

Regarding immediate root filling, if there is no periostitis he said to go through the foramen and fill immediately, as it is not necessary in most cases to wait any considerable length of time. In alveolar abscess we get suppuration and hyperplasia. After speaking of these and pus formation, he said to relieve the patient from severe pain, while opening up an abscessed tooth, to tie a strong cord to the neck of the tooth and request the patient to pull on this and not loosen the hold. If the pain is still felt, request him to pull a little harder, and keep this up until you have drilled through the foramen; and if you go beyond the apex it will do no harm.

He here spoke about disinfecting being carried to extremes, and that he had years ago used bichloride of mercury for disinfecting in the same manner as that of the present day, for all it is now used *scientifically*.

He said he did not go much on micrologists. He used to be one of them himself. A man that has been to camp-meeting and been converted six or seven times generally knows what he is talking about.

In regard to arsenic, or arsenious-acid, he said it has no affinity for the tissues and does nothing but take the place of the pabulum in the tissues.

*Dr. Watt:* After all, the point to be gained in practice is for a man to question himself and if he cannot answer his own questions and understand himself, he is not able to act.

Regarding the patient you should not be satisfied until you have inquired of his family history. Perhaps not satisfied with this you may inquire into other things such as constitution, habits, etc. If he has excessive habits as smoking, beer drinking, and the like, do not operate immediately. You must know all of these things before operating, if you want to be successful.

*Dr. Conrad:* I treat these teeth and in almost all cases fill immediately. After examination wash out the cavity with peroxide of hydrogen and go carefully to the end of the root, and when you know it is clean, fill it. When you find a molar with a calcarious deposit in the pulp chamber, you may think there is no opening into the root canal, but there is, and unless you go through this you will be liable to have trouble. When a root is filled to the apex perfectly with a hard substance, you should never have any trouble. My creed is to wash out the cavity thoroughly with warm water and then peroxide of hydrogen. Be cleanly, thorough, and open in to every root canal. Never let a cavity go unfilled where there is a bit of pulp tissue, and never let a root go until it is filled to the end.

*Dr. Jay:* The gentleman says never leave a portion of a root unfilled. I would like to inquire how he serves the molar roots?

*Dr. Conrad:* I cut through the crown, where I can, or even through the side just so I get into them. Perhaps we experience more trouble to find the opening in the anterior buccal root of the superior molar than any of the others. Where a root is tortuous, you must not drill straight or you will penetrate the side of the root and have trouble. It is best to go about one-half or two-thirds of the length of the root, then with a smaller Morey drill continue a little farther, then use Swiss broaches; keeping the canal flooded with peroxide of hydrogen. When this is carefully worked into the root the effervesence causes the infectuous material to be thrown out of the root toward the pulp chamber and not through the apical formen. Where there is a deposit in the chamber, and you cannot find the opening into the canals, place a little of the Herbst obtundent in the tooth and leave it until the next day, and if necessary repeat the operation until this covering is dissolved away and the opening is exposed.

*Dr. Sage:* Care should be used in the injection of medicaments into the substance of coagulated lymph or cellular tissue, for inflammatory action is liable to be set up. We often force the medicament through the root to the so-called pyogenic tract and get irritation and what we call over-treatment. I think it is not necessary to force any of this medicine through the root unless there is a sinus. The root canal should be kept flooded with antiseptics, and more precaution used if there is no fistulous opening. We should consider the question of diathesis of the patient, as there are certain symptoms we may recognize if we are observing. If a person has high cheek bones, is of stout build with a short neck, and has long bones, it points to a scrofulous diathesis. We have other indications of this in persons of a nervous or sanguine temperament, as those with red or sandy hair, light complexion, long slim necks, and flat chests. In these subjects immediate root filling is not advisable.

*Dr. Ames:* I believe that where we have no acute inflammation at the time, we can thoroughly remove the contents of the canals and fill immediately. In regard to iodide of potassium I have been in the habit of decomposing it by the galvanic current. Place the negative pole against the face of the patient, or let him hold it in the hand, and place the positive pole on the tissues where you require the decomposition of this substance. My method of disinfecting root canals is to take a Swiss broach, gold plate the point to prevent oxidation, attach to or hold in contact with the positive pole of the battery, and work it carefully and slowly to the apex of the canal, decomposing the iodide and thoroughly disinfecting. Then cleanse and fill the root immediately.

*Dr. Jay:* I have found nothing better than gutta-percha as a filling material for root canals.

*Dr. Taft:* There is wonderful unanimity in all these discussions. Every one has said he thinks that roots may be filled as soon as they are ready. This cannot be denied. We have a great variety of cases presented to us, and we must, as Drs. Watt and Atkinson have stated, take the accidental changes and conditions into account. If a patient presents himself and the surrounding circumstances are favorable, you can fill at once; but if a portion of pulp tissue or decomposed matter remain, it is not advisable to do so. Test this by placing a small pledget of cotton in the cavity and leave it for several minutes, then remove. If the odor of the cotton is offensive, and you close up the cavity, you will be liable to have trouble. If there is diseased tissue that needs to be disposed of at the end of the root, break it up. If this is not necessary, get the root into the best possible condition and seal with chlora-percha, being careful to avoid pressure. Care should be exercised in using carbolic acid. It it used too much. Apply the dam and use hot air as a desiccator. All these and other things should be taken into account before filling.

*Dr. Fletcher* had a failure to report. A man who had taken whisky, and perhaps quinine, had a superior right lateral that had been sore for five years but had given no particular pain. It needed refilling, and in taking out the filling he found the pulp chamber empty. There was a slight odor and soreness. He used peroxide of hydrogen, as Dr. Conrad suggested, carefully filled the tooth, and dismissed him. The next day he returned suffering from the effects. He punctured the alveolus, got a discharge of blood which relieved the patient somewhat, but to-day that man is in bed.

*Dr. Sage* spoke of peroxide of hydrogen, its action, etc. He then referred to the treatment of root canals with chloride of zinc to coagulate the albuminous materials.

*Dr. Atkinson:* When treated in this way, hydro-chlor-zincate-of-albumen is formed. On removing this from canals, I have found it to be as clear as glass, and have said that I did not know of a more perfect filling, but how long it would remain in this condition I do not know. He spoke of gaseous bodies penetrating the tissues, and that he had seen blood abscesses which required a long treatment and gave all the symptoms of necrosis. Such cases usually succumb to a solution of salt and water followed by applications of aromatic sulphuric acid. In treating diseases where the system needs toning up, he had found McKesson & Robbin's preparation of nux-vomica and phosphorus cantharadies of value. Take a two-grain capsule of sulphate of cinchonidia twice a day, and one pill of the nux-vomica preparation at night.

This gives the system tone, and then your other medicines will take hold.

When a pus abscess is treated with peroxide of hydrogen, we get a creamy mixture and effervescence. You can prove the action by applying a heated instrument. If carbonic acid is generated, the instrument is colored black; if oxygen is present, the heat is increased; and if you get a flame, it indicates the presence of hydrogen.

